# A Comprehensive Review on Harnessing Soy Proteins in the Manufacture of Healthy Foods through Extrusion

**DOI:** 10.3390/foods13142215

**Published:** 2024-07-14

**Authors:** Yuyang Huang, Linlin Liu, Bingyu Sun, Ying Zhu, Mingshou Lv, Yang Li, Xiuqing Zhu

**Affiliations:** 1 College of Food Engineering, Harbin University of Commerce, Harbin 150028, China; huangyuyang1979@163.com (Y.H.); keaiduolinlin@126.com (L.L.); sby0451@163.com (B.S.); 13258512068@163.com (Y.Z.); lumingshou@hrbcu.edu.cn (M.L.); 2 College of Food Science, Northeast Agricultural University, Harbin 150030, China; yangli@neau.edu.cn

**Keywords:** soy protein, extrusion, low-calorie, health-promoting foods

## Abstract

The global development of livestock production systems, accelerated by the growing demand for animal products, has greatly contributed to land-use change, greenhouse gas emissions, and pollution of the local environment. Further, excessive consumption of animal products has been linked with cardiovascular diseases, digestive system diseases, diabetes, and cancer. On the other hand, snacks, pasta, and bread available on the market are made from wheat, fat, salt, and sugar, which contribute to the risk of cardiovascular diseases. To counter these issues, a range of plant protein-based food products have been developed using different processing techniques, such as extrusion. Given the easy scalability, low cost of extrusion technology, and health benefits of soy proteins, this review focuses on the extrusion of soy protein and the potential application of soy protein-based extrudates in the manufacture of healthy, nutritious, and sustainable meat analogs, snacks, pasta products, and breakfast cereals. This review discusses the addition of soy protein to reformulate hypercaloric foods through extrusion technology. It also explores physical and chemical changes of soy proteins/soy protein blends during low and high moisture extrusion. Hydrogen bonds, disulfide bonds, and hydrophobic interactions influence the properties of the extrudates. Adding soy protein to snacks, pasta, breakfast cereals, and meat analogs affects their nutritional value, physicochemical properties, and sensory characteristics. The use of soy proteins in the production of low-calorie food could be an excellent opportunity for the future development of the soybean processing industry.

## 1. Introduction

The food industries produce a large amount of ultra-processed food which requires minimum cooking and preparation time. Various consumers like these food products because of their excellent sensory properties. Ultra-processed foods have a significant amount of fats, refined carbohydrates, sugars, and salts; hence, their consumption may play a negative role in the development of cardiovascular diseases [1]. Food reformulation is required to help alleviate the problems linked with the excessive consumption of ultra-processed containing high sugar, fats, and refined carbohydrates. In general, the reformulation of food products attempts to reduce the presence of components with health side effects and increase the presence of valuable compounds. Soy proteins have shown various functional and physicochemical properties; therefore, they can provide solutions to counter the challenges of hypercaloric food reformulation. Soybean flour, soy protein concentrates, and soy protein isolates are commercially available soy protein products obtained using various techniques. The composition and extraction process influences the functional properties of the protein during processing.

Extrusion is a complex technology that involves mixing, mechanical shearing, and pressurization to generate the desired shape and texture. Based on this treatment, various variables such as temperature, moisture, raw material composition, viscosity, and mass flow are paramount to yielding extrudates with good physicochemical properties [2]. Extrusion technology is preferably used to prepare meat analogs, which are plant proteins with a fibrous texture similar to that of real meat. Soy protein meat analogs and other extruded soy products can be used as meat alternatives or in the formulation of products with high protein content, as it has been previously reported that the excessive consumption of red meat may lead to cardiovascular diseases [3]. Also, the adoption of textured plant protein prepared using extrusion technology may significantly decrease the environmental impact linked with animal husbandry.

In recent years, various authors have summarized the structural modification of plant proteins and the development of food products using extrusion technology [4,5,6,7]. However, no up-to-date review proposes adding soy protein to the food matrix to reformulate hypercaloric, high-cholesterol foods through extrusion technology. Extruded soy proteins are outstanding materials for absorbing flavors, colors, spices, and other seasonings, which endow their derivative products with exceptional sensory properties. Given the consumer demand for healthy and nutritious food products, this review presents the addition of soy protein in the manufacture of snacks, meat analogs, bread, pasta, and breakfast cereals through extrusion. Adding soy proteins in product formulation may improve nutritional value and enhance public health, which could be an excellent opportunity for the soybean processing industry. Further, over the last years, there has been a need for shifting to a plant protein-based diet; these products would respond to consumer expectations.

## 2. Soy Protein Properties and Biological Functions

### 2.1. Composition

The major commercially available products of soy proteins are soybean flour, soy protein concentrates, and soy protein isolates. The protein concentration is approximately 50% in soybean flour, 70% in soy protein concentrate, and 85- 90% in soy protein isolate [8]. The concentration of soy protein products depends on the processing conditions, impurities, and refining process. Soybean consists of two major proteins: albumins and globulins. Taking into account the solubility, albumins are water-soluble, and globulins are salt-soluble. Soy proteins can be separated by centrifugation into 2S, 7S, 11S, and 15S fractions [8]. The 2S ranges from 8 to 22% of the extractable proteins, β-conglycinin accounts for 35%, 11S named glycinin accounts for 31–52% of soluble proteins, and 15S accounts for 5% of soluble proteins [9]. The 11S globulins possess a molecular weight of approximately 360 kDa; the 7S globulin molecular weight ranges from 50 to 170 kDa, and the molecular weight of albumins can vary from 5 to 80 kDa [10].

### 2.2. Biological Functions

Soy proteins contain most of the essential amino acids necessary for the human body. Due to the structure and amino acid and peptide composition, soy proteins may have different effects on health in children, young adults, and old individuals [11]. A previous study involving soy proteins demonstrated that soy proteins can improve the cholesterol-lowering effect after an in vitro simulated digestion [12]. It was previously suggested that high soy protein ameliorates the body composition in overweight and obese individuals by decreasing fats and keeping muscle [12]. A study also reported that soy protein supplementation can improve the lipid profile among healthy people [13]. Soy proteins also can affect the marker of bone metabolism and lipid metabolism in males and females aged between 27 and 87 years [14]. The findings showed that soy protein may retard the progression of diabetic nephropathy and improve renal function markers [15]. Soy proteins and peptides can significantly prevent breast cancer development [16]. The results of Kovacs-Nolan et al. [17] demonstrated that soy proteins can prevent the production of pro-inflammatory mediators in intestinal epithelial and immune cells and significantly decrease the severity of colitis. The effect of soy proteins on atherosclerotic lesions was examined, and the findings revealed that soy protein isolates may decrease atherosclerotic lesions and prevent macrophage infiltration [11]. Dietary soy protein isolates may lower the level of luminal secretory immunoglobulin A and can also attenuate the production of intestinal mucin [18].

The health benefits of soy proteins are attributed to the ability to reduce the activity of various enzymes, mRNA expression, and different biomarkers involved in metabolic pathways. The composition and molecular weight of soy protein peptides play an important role in regulating and inhibiting gene expression. The 7S and 11S fractions with at least one glutamine Q residue and a molecular weight ranging between 5 and 10 KDa have shown high anti-inflammatory and anticancer effects. Further, the presence of serine residue in soy protein peptides also correlated with high anticancer and anti-inflammatory. Soy protein peptides may prevent the excessive accumulation of free radicals and inflammation, control body balance, and decrease illness associated with excessive free radical accumulation.

## 3. Challenges in the Utilization of Soy Proteins

Soy proteins are widely used in food processing technology to fabricate a wide range of products. However, soy protein’s composition, natural flavor, and physicochemical properties can negatively influence its potential utilization and consumer acceptability. This part briefly introduces the adverse effects and challenges of soy protein samples during extrusion.

### 3.1. Deficiency and Loss of Amino Acids

The utilization of soy protein-based products is limited by a deficiency of sulfur amino acids, which can influence their nutritional value. Sulfur amino acids (cysteine and methionine) play an important role in preventing different diseases and performing biological functions. Methionine is an essential amino acid and may act on various cellular functions, while cysteine is a conditionally essential amino acid because the body can convert methionine into cysteine [19]. It was previously reported that cysteine is a precursor of glutathione, taurine, and H_2_S, playing an important role in intracellular antioxidant defense, cardiovascular function, and immunomodulatory [20]. Sulfur-containing amino acids are indispensable in human nutrition. Some strategies, such as blending soy proteins with corn proteins or other good sources of these amino acids, are necessary to increase the nutritional value of soy protein-based products.

During soy protein processing, they are exposed to thermal, chemical, and mechanical treatments that can induce conformational characteristics and affect their stability and functionality. For example, subjecting soy protein isolates to high temperatures, high pressures, and high shear forces can cause protein refolding, the modification of side amino acid chains, and increased carbonylation, suggesting that these samples are susceptible to oxidation during storage [21]. Further, protein oxidation can lead to additional crosslinking, backbone fragmentation, and conformation change [22]. It is assumed that the presence of other macromolecules can govern the aggregation rate and decrease the extent of protein oxidation during storage.

### 3.2. Beany and Grassy Flavor

Soy protein products may possess an unacceptable beany and grassy flavor, significantly affecting consumer acceptability. The beany flavor is a mixture of many compounds generated during the growth and processing of soybeans. These compounds consist of fatty aldehydes, fatty alcohols, fatty ketones, furans, furan derivatives, and aromatic compounds, with hexanal being the most important one [23]. The sensory properties of soy protein-based bread showed that non-treated samples have a beany flavor, resulting in low sensory properties and poor consumer acceptability [23]. The consumer rejected soybean beverages due to the intense beany and grassy flavor [24]. The threshold of the beany and grassy flavor influences the sensory characteristics and consumer acceptability. A significant decrease in the beany and grassy flavor using processing methods or mixing with other ingredients can increase the acceptability of soy protein-based products.

Three different methods are widely used to remove soybean’s beany and grassy flavor, including the physical method, chemical method, and biotechnological method [12]. Physical methods such as pH adjustment to inhibit the activity of lipoxygenase and the application of organic solvent during protein extraction can effectively remove the beany flavor. Genetic engineering, enzymatic treatment, fermentation, and germination are biotechnological techniques used to remove beany flavor and improve the sensory properties of soybean products. Physical methods include conventional heating, gamma irradiation, pulse electric field, use of flavor enhancers, and extrusion aromatization. High-temperature treatment is the most common method used to improve the soy protein flavor. During soy protein extrusion, samples are subjected to high temperatures, which may decrease the grassy and beany flavors. In a study involving the treatment of soy proteins [12], the results showed that heat exposure may decrease the volatile components responsible for the grassy and beany flavor by 80%. Additionally, high-temperature treatment of soy proteins in the presence of carbohydrates can lead to the formation of Maillard reaction products that may effectively mask the unpleasant flavor of soy proteins [24]. It was demonstrated that high temperature (110–120 °C) and short-time treatments (approximately 10 min) may significantly decrease the off-flavor and preserve the nutritional composition of the resultant soy milk [25,26], suggesting that the selection of appropriate heating conditions is paramount to removing unpleasant flavor while retaining the physicochemical properties of soybean products. In attempts to improve the flavor and acceptability of extruded soy protein isolates, soy protein isolate was mixed with various flavor enhancers such as monosodium glutamate and disodium inosinate [27]. It was found that the addition of flavor enhancers increases the density, reduces the cutting force, and increases the compression force of rehydrated soy protein extrudates. The extrudates have shown high aroma intensity and high overall acceptability. It was concluded that the high viscosity and low mobility of molecules during extrusion may enhance the interaction between soy protein isolates and flavor enhancers, resulting in products with excellent sensory properties. As both 11S and 7S denature during extrusion cooking ≥ 100 °C, it should be noted that in the treatment at 80 °C, 7S fully denatures while 11S is partially denatured.

## 4. Extrusion of Soy Proteins

**Figure 1:**
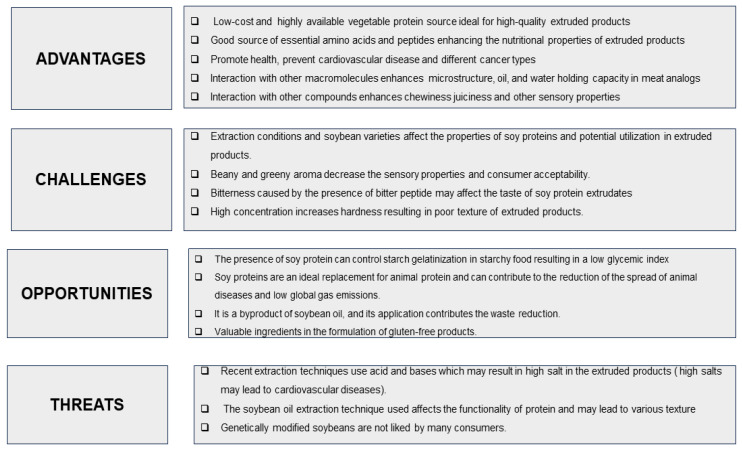
Analysis of the use of soy protein in extrusion.

Extrusion is an adaptable technology that combines thermomechanical operations such as homogenizing, heating, kneading, shearing, and molding [28]. Extrusion is a multifaceted and cost-effective technique widely used in the food and feed industries [28]. Extrusion technology can be used to alter the conformation characteristics of soy proteins and improve their biological functions. Further, this technique can be performed continuously with high input. It has been demonstrated that the temperature and shear conditions applied inside the extruder can impact the interaction of sample constituents [29]. The results have shown that increasing the mechanical energy can enhance the extent of protein breakdown into small fractions, suggesting that mechanical shear may lead to the dissociation or depolymerization of proteins [30]. Furthermore, temperature affects the water absorption, solubility, color, and expansion of the extrudates [27]. Single and twin screws are the major extruders widely utilized in food industries. A single-screw extruder uses one rotating screw inside a fixed barrel, while a twin-screw consists of two counter-rotating screws moving inside a barrel [31]. The advantage of a single-screw extruder is linked with a low cost, while the advantages of twin-screw extruders are associated with handling high-capacity and complex raw materials. Extruder components such as screws, barrels, and dyes are used to generate unique finished products. Depending on the use of the final product, extrusion can be conducted at a high or low moisture. Extrudates prepared at low moisture (below 35%) have shown a sponge-like structure and may require rehydration before use as a food ingredient [32]. On the other hand, extrudates produced at a high moisture possess a dense structure and strong elasticity, and they can be directly consumed [33]. Figure 1 shows the analysis of the use of soy protein in extrusion.

### 4.1. Low Moisture Extrusion

Low moisture extrusion (moisture below 30–35%) is a technique that uses a high temperature and shear to generate a wide range of food products. During low moisture extrusion, raw materials can undergo physical and chemical transformations that may lead to the production of a wide range of products. This technique may be used to manufacture healthy snacks, noodles, etc. Feed moisture, temperature, screw speed, viscosity, and feed rate significantly affect the characteristics of the extrudates. The following part will focus on the low moisture extrusion of soy proteins (Table 1).

Soy protein concentrate was subjected to low moisture extrusion at feed moisture and temperature of 18–25% and 110–160 °C, respectively [34]; the results indicated that the extrusion of soy protein at 25% enhanced the denaturation, aggregation, and crosslinking degree. This was attributed to the formation of large aggregates via hydrogen bonds at 25%; however, it was demonstrated that a moisture of 18% favored the dissociation of soy protein aggregates. Additionally, the dissociation of large aggregates at 18% improved the solubility. Larger aggregates produced at 25% demonstrated high hydrophobicity and were attributed to the aggregation that buries hydrophilic amino acids. The effect of low moisture extrusion at 8–12% on the quality of the extrudates was investigated. It was demonstrated that products extruded at a low moisture level of 8% were less resilient, had increased air cell size, and were less uniform, which may lead to fragile extrudates during handling [45]. On the other hand, increasing the feed moisture to ≥12% can result in products with a fine cell structure with less fissuring and less susceptibility to breakage, which may be due to structure, plasticity, and elasticity change [46]. On the other hand, increasing the temperature from 110 to 130 °C decreased soy protein hydrophobicity and decreased solubility, and the particle size increased. However, further temperature increases from 130 to 160 °C decrease the particle size and increase the hydrophobicity, and the solubility of soy proteins increase, which may indicate a partial dissociation of aggregates generated at 130 °C. Soy protein isolates were exposed to low moisture extrusion processing with 30% feed moisture at 170 °C [27]; the findings demonstrated that the addition of volatile and flavor compounds decreased the viscosity, which favored the interaction of protein and volatile compounds. The protein and volatile interaction improves the density of extrudates and increases the compression force of rehydrated samples. The processing time, temperature, and moisture content significantly affected the retention of volatile compounds and the sensory evaluation. Effects of particle size and extrusion parameters on soybean meal (rich proteins) were studied by Singh and Koksel. [31]. The results showed that adjusting the particle size of raw materials affects the melt viscosity, the rate of heat transfer, and the mechanical energy required during extrusion. Also, changing the barrel temperature from 110 to 150 °C increased the nitrogen solubility, gel-forming capacity, and emulsion stability of soy protein extrudates. Additionally, the results confirmed that increasing the feed moisture from 15 to 27 g water/100 g enhances the water holding capacity, emulsion capacity, and stability of extrudates. Excessive high temperatures ≥ 150–170 °C can lead to the formation of soy protein insoluble aggregates, resulting in the loss of rigidity of the extrudates [31]. High temperatures ≥ 150–170 °C also affect the retention of moisture and can lower the rehydration behavior of soy protein extrudates. High temperatures can lead to the production of dense and hard extruded soy proteins with low bulk density and poor expansion [31]. This can be attributed to high-temperature treatments that may break the quaternary structure of 11S into fractions, which may later generate insoluble aggregates [47]. Further, subjecting soy protein to extreme temperatures can destroy some amino acids and significantly influence the texture [34]. The purity of soy proteins is another important factor that may determine the quality of extrudates; the presence of other molecules can affect the protein–protein interaction, aggregation rate, and texture of the finished soy protein extrudates.

### 4.2. High Moisture Extrusion

High moisture extrusion is a process used to texture plant proteins into fibrous textured products. During high moisture extrusion, the feed moisture ranges between 40 and 80% [48]. The moisture content used during high moisture extrusion depends on the temperature and type of ingredients in the formulation. Feed moisture, temperature, pressure, feeding speed, and mechanical input have a considerable effect on the properties of textured soy protein. The following section will emphasize the high moisture extrusion of soy proteins (Table 1).

The effect of the specific mechanical energy (SME) on the properties of textured soy protein isolates processed at a high moisture extrusion of 50% and a screw speed of 150 rpm was investigated [49]; it was pointed out that changing (SME) from 819.70 to 1258.70 kJ/kg resulted in low lightness, and high hardness and tensile strength. The viscosity of the dough decreased when the SME increased. Further, a high SME (1258.70 kJ/kg) significantly increased the solubility of proteins and the formation of low molecular weight soy protein subunits. These findings may be attributed to the different mobility of soy protein components at different processing conditions. Also, the change in soy protein molecular interactions may be responsible for the physicochemical changes during extrusion processing. The influence of soy protein concentrate extrusion on protein–protein interactions during a high moisture extrusion of 60%, screw speeds of 180 rpm, 500 rpm, and 800 rpm, and specific mechanical inputs of 85–350 kJ/kg were studied [38]. The results showed that subjecting soy proteins to ≥500 rpm and ˃140 °C drastically decreased the extruder pressure and the melt viscosity, which may be attributed to the degradation of soluble/insoluble macromolecules, the decrease in molecular weight, and the change in molecular structure. Su et al. [50] also reported the physical and chemical properties of soy protein isolates treated with sodium sulfate during high moisture extrusion. The results revealed that the pre-treatment of soy protein with sodium sulfate (1.5–3%) partially denatured the protein and significantly reduced the aggregates in the extrudates. The pre-treatment of soy protein with sodium sulfate (1.5–3%) increased the solubility and surface hydrophobicity but decreased the water absorption capacity. This was attributed to the role of sodium sulfate in breaking the disulfide bonds and decreasing the specific units in protein molecules. Another study by Wang et al. [36] discussed the high moisture extrusion of soy protein isolates using a feed moisture of 60%, a feed rate of 0.3 kg/h, a screw speed of 200 rpm, and temperatures of 140 and 160 °C. Their results demonstrated that soy protein isolates can yield less fibrous extrudates with more compact structures beneficial to the formation of a harder rubber-like texture. The presence of insoluble and non-melting components promoted the formation of a second phase and yielded fibrous structures.

During high moisture extrusion, the amount of water in the matrix positively correlates with the reaction rate. It was pointed out that increasing the moisture content can significantly decrease the viscosity and increase soy protein mobility, resulting in a high reaction rate during extrusion [51]. On the other hand, decreasing moisture content during extrusion may prevent the ongoing interaction between soy proteins. Temperature is another important variable during high moisture extrusion as it monitors from the melting’s beginning to the end to guarantee that melting, conformation change, and protein texturization are fully achieved [52]. At elevated temperatures, denaturation, polymerization, and aggregation reactions lead to the crosslinking and texturization of soy proteins. In addition, cooling rate and temperature can influence a textured protein’s expansion. A low cooling rate may also influence the elasticity and strength of textured proteins [53]. Components such as carbohydrates and sugars can affect the alteration degree of soy proteins. The presence of carbohydrates and sugars during extrusion results in the formation of Maillard reaction products, which may lead to a more ordered soy protein in final extruded products [54]. Additionally, change in specific mechanical energy is crucial to achieving high-quality and reproducible soy protein extrudates. It was pointed out that specific mechanical energy can influence the aggregation rate and alter the conformation of the protein, which may impact the texture and sensory properties of the extrudates [49]. The screw speed affects the water absorption, water solubility, and the rate of transformation of soy protein during the extrusion process [55]. The physicochemical properties of raw materials, extruder settings, and process variables are important to generating high-quality extrudates.

## 5. Extrusion of Soy Protein-Based Blends

### 5.1. Low Moisture Extrusion of Soy Protein-Based Blends

**Figure 2:**
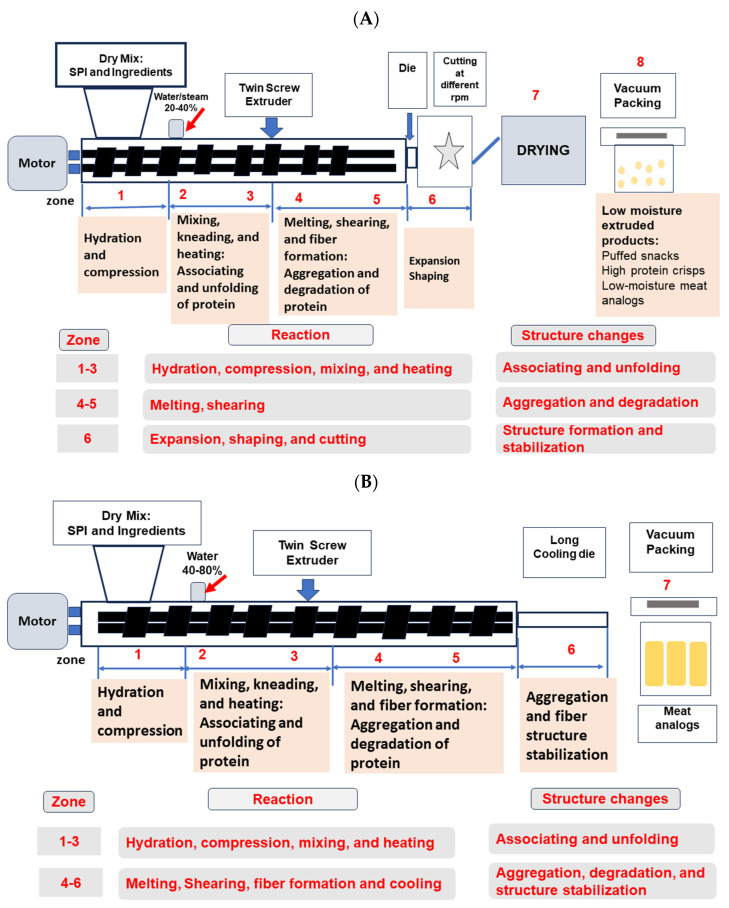
Diagram of twin screw-extruder. (**A**) Low moisture extrusion of plant protein-based materials. (**B**) High moisture extrusion of plant protein-based materials.

Soy proteins are blended with other plant proteins/polysaccharides to ameliorate the expansion capacity of the melt and increase the water-holding capacity. Soy proteins are also mixed with other proteins to improve the content of amino acids, such as lysine and sulfur-containing amino acids [56]. Blending soy protein with other ingredients can influence the porosity, microstructure, color, texture, and flavor of the final products. Raw material composition, ratio, concentration, feeding speed, moisture content, temperature, and screw speed can affect the quality and storage stability of the extrudates (Table 1). Figure 2A indicates low moisture extrusion of soy protein-based products.

Ahmad et al. [56] examined the low moisture (20–30%) extrusion of soy protein concentrate and fish protein concentrate blends. Their findings showed that the extrudates possessed a high nutritional value, excellent porosity, even pore distribution, and homogenous cell wall thickness, which were advantageous for oil adsorption. These results can be associated with the concentration of the fish protein concentrate (FPC) that enhanced the plasticization and intramolecular binding forces. In another study, soy protein and corn were subjected to low moisture extrusion using a feed moisture of 20–30%, 30–140 °C, a screw speed of 200–400 rpm, and a feeding of 20 kg/h. It was revealed that extrusion at an appropriate moisture and temperature can destroy the immunoreactivity of the allergenic protein, thereby resulting in a decrease in the removal of allergens [35]. Further, changing the moisture from 20% to 40% during extrusion led to a reduction of specific volume and a significant decrease in water holding capacity, texture, and color. These may be attributed to the critical role of water in the expansion process during extrusion processing. Water affects the gelatinization process, cell structure, and mechanical properties [27].

In addition to fish proteins, other proteins are used for dough preparation, such as wheat protein, corn protein, and starch, to improve the nutritional value and modify the physicochemical properties of the resultant extrudates. During the extrusion of soy protein and high amylose corn starch, soy protein altered the expansion, water holding capacity, texture, average size, cell thickness, and distribution [35]. Further, the gelatinization degree and thermal and mechanical degradation of starch in the mixture affected the characteristics of the extrudates, such as texture, hardness, and shape [57]. High temperatures and mechanical forces used during extrusion induce protein denaturation, which in turn can change the structure and lead to various interactions. It was suggested that polysaccharides, such as alginate, guar gum, and xanthan gum, could increase the texture and enhance the stability of soy proteins during processing and storage [58]. This may be attributed to the soy protein and polysaccharide interconnection, which can increase the network strength and mechanical properties, resulting in greater stability. On the other hand, it was demonstrated that non-covalent and covalent (disulfide) interactions between soy protein and wheat gluten may promote the formation of more alpha-helices, which can affect the texture and porosity of the final products [41].

### 5.2. High Moisture Extrusion of Soy Protein-Based Blends

Soy proteins are used to generate textured plant proteins; however, the utilization of soy proteins alone is linked with some limitations, such as yielding products that have poor taste with a dense and hard texture [59]. Proteins are blended with other macromolecules to improve the techno-functional and sensory properties of high moisture extrudates. Studies have previously revealed that the partial replacement of soy proteins with wheat proteins can enhance the texture, chewiness, cohesiveness, and cutting strength [48]. Further, the addition of carbohydrates can facilitate the extrusion process and increase the physicochemical properties of the extrudates [60]. Figure 2B indicates high moisture extrusion of soy protein-based products. The effect of processing conditions and raw material physicochemical properties on the high moisture extrusion of soy protein blends are displayed in Table 1.

A soy protein concentrate was blended with wheat gluten at different ratios (SPC/Wheat gluten 89:0, 79:10, 69:20, and 59:30) and starch to prepare high moisture extrudates [41]. The formation of fibrous extrudates was due to various interactions, including hydrogen bonds, disulfide bonds, hydrophobic interactions, and their combinations. It was demonstrated that reducing the ratio of SPC to wheat protein enhanced the fibrous formation, texture, and sensory properties. These findings concluded that 59% soy protein, 30% wheat gluten, and 2.7% starch can yield extrudates with the highest fibrous structure, hardness, and chewiness. The effect of iota carrageenan at 0.75, 1.5, 2.25, and 3% on the properties of soy protein extrudates was investigated [42]. The addition of iota carrageenan drastically increased the viscosity inside the extruder. Samples also demonstrated a high water-holding capacity attributed to compact soy protein–iota carrageenan, which prevented water release from the matrix. The compact structure generated by the soy protein–iota carrageenan caused a relatively low water uptake that may result in a low cooking yield. Weak or strong, specific or nonspecific, attractive or repulsive interactions are engaged in the network generation between soy protein and iota carrageenan. It was confirmed that 1.5% of iota carrageenan was ideal to produce better physical and sensorial properties. The authors Kiiru et al., 2020 [61] prepared soy proteins and cricket flour blend ratios (Soy proteins/cricket flours 85:15, 70:30, and 55:45); it was demonstrated that low-fat cricket flour ranging from 15 to 30% can create distinct and dense anisotropic structures. On the other hand, full-fat cricket flour generated a stiff texture attributed to the presence of lipids during extrusion. The boiling of oil enhances the heat and mass transfer that leads to the evaporation of water in the matrix, resulting in extrudates with hard external layers. Mixing an ideal concentration of cricket flour and soy protein isolate may lead to extrudates with dense fiber networks and tensile properties.

During high moisture extrusion of soy protein-based blends, the temperature directly influences the texture of meat analogs. The findings showed that an inlet temperature of 120 °C does not induce the formation of the fiber-like structure while increasing the inlet temperature to 160 °C results in the formation of a lamella structure [62]. The blends of soy proteins and wheat proteins are widely utilized in textured protein and meat analog production. Wheat proteins have demonstrated a high binding capacity, dough-forming capacity, leavening capacity, and viscoelastic behavior [48]. However, the use of wheat protein in meat analogs is restricted by people with high sensitivity to gluten. The blends of soy proteins and plant proteins such as pea protein, corn protein, mung bean protein, and peanut protein are used in the formulation of meat analogs to tackle the drawbacks linked with wheat proteins. It was noted that some plant proteins (peanut protein and mung bean protein) produce weak gel and poor texture properties [63]. On the other hand, polysaccharides are added to soy protein during meat analog manufacturing due to their functional properties such as thickening, gelling, and rheology modifier, and their addition can potentially enhance the texture, chewiness, and water absorption capacity [64]. It was demonstrated that the addition of carrageenan, sodium alginate, and starch may alter the fibrous structure of meat analogs by enhancing plant protein aggregation during high moisture extrusion [52]. For example, the findings showed that the addition of 6% sodium alginate can improve the rehydration and digestion rate of soy protein-based extrudates, while carboxymethylcellulose sodium 6% produces extrudates with low chewiness and a compact structure [65]. The blends of soy protein concentrate and iota carrageenan revealed a compact structure and low chewiness compared to sodium alginate and carboxymethylcellulose sodium [65]. Extrusion of soy proteins and polysaccharides favors hydrogen bonds and hydrophobic interactions. These interactions can cause a significant change in the ratio of protein subunits. Polysaccharides also enhance the fragmentation of the native protein from a high molecular weight to a low molecular weight, which may consequently be reconstructed in large networks [54]. Most polysaccharides influence the formation of fibrous structures through enhanced protein aggregation during extrusion processing. The type of extruder, screw configuration, screw diameter, number of barrel zones, and cooling dye require strict monitoring to generate high-quality meat analogs.

## 6. Potential Application of Soy Protein and Soy Protein-Based Blends in Healthy Food Formulation through Extrusion

The incidence of cardiovascular disease, cancer, diabetes, and obesity has increased due to the excessive consumption of hypercaloric food and animal products [66]. There is also evidence that the animal production system increases global greenhouse gas emissions and enhances the generation of wastes that can affect human health and the environment [67]. It has been proposed that the substitution of animal products with plant-based diets can significantly decrease the environmental impact and decrease the rate of non-communicable diseases. For example, studies have suggested that soy protein can lower blood cholesterol and blood pressure, adjust blood sugar levels, and significantly decrease the development of certain cancers [68].

### 6.1. Application in Meat Analogs

Meat analogs are textured plant proteins that possess a texture, mouthfeel, taste, and nutritional value comparable to that of meat products [69]. Extrusion technology can modify plant proteins into fibrous products. Due to its availability, low cost, nutritional value, and health benefits, soy proteins are widely used to prepare meat analogs. Table 2 displays soy protein and soy protein-based blends of meat analogs.

Soy protein isolates, wheat glutens, and natural flavors were used to produce meat analogs [60]. The results demonstrated that a high concentration of wheat gluten in the formulation and a high moisture content influenced the microstructure, the water-holding capacity, protein structure, and retention of volatile compounds. The high molecular weight of wheat gluten subunits can easily form aggregates and increase the viscosity of the molten during the extrusion process, leading to more fibrous structures. The three-dimensional architecture created by protein aggregation favored the high retention of volatile compounds in meat analogs. Soy protein isolate, wheat gluten, and starch at a ratio of 5:4:1 were used to form meatless burger patties, and the results showed that extrusion processing at 40–50% moisture and 130–150 °C can generate meat analogs similar to a commercially available meat patty [70]. Also, changing the moisture content from 40% to 50% resulted in high springiness, cohesiveness, hardness, and cutting strength, while augmenting the temperature from 130 to 150 °C decreased springiness, cohesiveness, and hardness. The observed changes in springiness, cohesiveness, and hardness were attributed to the formation of a much stronger protein network. In another research study, meat analogs were prepared using soy protein concentrate fortified with free or encapsulated iron [22]. The presence of free or encapsulated iron did not impact the texturization process. However, the presence of iron decreased the solubility, which may be linked with protein oxidation. During the texturization of plant proteins, the results indicated that the incorporation of encapsulated iron can affect the oxidation rate of meat analogs during storage.

The concentration of soy proteins and the composition of various ingredients affect the overall quality of meat analogs. A change in soy protein and other ingredient ratios can influence the fibrous structure, hardness, chewiness, and cooking yield of soy-based meat analogs [78]. A moisture content of around 50–60% is optimal for generating meat analogs with similar meat-like fiber structures; however, a further increase in the moisture may significantly influence the hardness, chewiness, and aggregation rate [79]. With a further increase in moisture (60–90%), the layered fibrous structure of meat analogs disappeared due to the high fluidity of the material and a significant decrease in shear strength and pressure. A temperature between 120 and 140 °C is recommended to induce conformation change and intramolecular bonding to generate a meat-like product structure. It should be noted that the concentration of proteins should be kept above 35% to produce meat analogs with excellent physicochemical properties, cooking yield, and ideal sensory properties [79].

Comparing meat analogs with meat products, meat analogs have demonstrated advantages such as a relatively high viscosity in the stomach, a high protein hydrolysis, and the inhibition of fat hydrolysis during in vitro digestion [80]. Individuals following a plant-based protein diet demonstrated an important decrease in total fat and saturated fat than individuals fed with an animal protein-based diet [81]. It was suggested that the consumption of soy protein-based meat analogs may assist in the prevention of some chronic diseases. For example, meat analogs have a low energy value, low concentration of saturated fats, and high concentration of dietary fibers, which make these products healthier. The main drawback of soy protein-based meat analogs has been linked with the texture. Also, plant protein does not naturally appear in a fibrillar orientation such as myofibrillar and myoglobin in muscle, and this may render the process of plant protein extrusion to obtain similar architecture very difficult [82]. Also, the replication of meat juiciness and sensory characteristics using soy protein-based meat analogs remains another challenge [3]. On the other hand, the use of 15% oil or fat during meat analog production can induce lubrication of the matrix that can prevent fiber alignment [83]. The addition of a high concentration of oil/fats renders the material lubricated, which may negatively affect the shearing forces during extrusion processing. As fats/lipids are used to improve the taste of meat analogs, they should be carefully selected and added to the matrix. It is therefore recommended that the extrusion parameters, ratios, and concentrations of ingredients should be studied and optimized to generate soy protein-based meat analogs similar to meat or meat products for the intended use.

### 6.2. Application in Snack Products Formulation

Snacks are various small amounts of food, including dried fruits, jellies, chocolate, potato chips, candies, etc., widely consumed between meals. Due to their salty and sweet taste, these types of food are preferred among various consumers, including children and adolescents. While the lifestyle is changing in modern society, consumers are shifting from a full meal to snacks. Most snacks sold in supermarkets are rich in fats, salts, sugars, and highly digestible starches and are low in nutritive value [84]. It has been reported that the excessive consumption of snack food can increase the risk of obesity, type-2 diabetes, and other cardiovascular diseases [85]. Skoczek-Rubinska and Bajerska. [86] showed that the consumption of high-energy snacks can contribute to more than 40% of daily energy intake, suggesting that the addition of energy intake may lead to weight gain. Therefore, to reduce the risk linked with high-energy snacks, there is a need to produce healthy and nutritious snacks using extrusion technology. Soy protein/soy protein blends can be utilized to prepare high protein value snacks, which can allow the achievement of the recommended daily intake of proteins in different consumers.

Soy proteins, carrot pomaces, rice flours, and cauliflower trimmings were subjected to low moisture extrusion to generate highly nutritious ready-to-eat snacks [87]. It was pointed out that the extrudates possessed a 10.2% protein content and 0.85% fiber content with a high consumer overall acceptability of 76%. These snacks can be recommended to children and adults as healthy snacks. Sprouted soybeans cultured in FeSO_4_ and corn were used to prepare low moisture extrudate snacks, and it was pointed out that approximately a 35 g portion of this product can sustain 50% of the recommended daily intake of iron [88]. Extruded snacks were prepared by mixing defatted soy flour, barnyard millet, Indian gooseberry, and rice flour [89]. The results revealed that these snacks can reach a protein content of 19%, iron content of 15 mg/100 g, and high consumer acceptability, suggesting that these snacks can be recommended to prevent anemia. Defatted soy flour and banana extrudates were used to prepare a healthy snack that can potentially be used by consumers with an inability to digest gluten [74]. It was found that the mixture of soy and banana flour can be used to make low glycemic and nutritious snacks with high protein, resistant starches, dietary fibers, and phenolic compounds.

Ingredients selection, process control, shape, color, and taste affect the consumer acceptability of extruded soy protein-based snacks. As protein is the major ingredient of extruded soy protein snacks, moisture content, aggregation rate, intramolecular bonding, and conformational change during extrusion will impact the quality of the finished products [28]. Some ingredients, such as starch or starchy ingredients, are mixed with soy protein during extrusion, and van der Sman and Broeze [90] reported that extrusion can enhance starch gelatinization. The physical and chemical changes of soy protein and other ingredients affect the porosity, density, shaping, and sensory characteristics of snacks. The characteristics of each ingredient, the interaction, and the processing conditions contribute to the development of different flavors and their precursor, which in turn can favor consumer acceptability [91].

In comparison with other snacks, soy protein-rich snacks have demonstrated advantages such as low calories and a high protein value. Considering the health benefits of soy products, including the prevention of coronary heart disease, diabetes, and colon cancer, these soy protein extrudate snacks can be suitable for children and adults [84]. Starch is among the major ingredients used in snacks. It has been demonstrated that extrusion increases starch digestibility and the risk of a high glycemic index [92]; thus, the partial replacement of starch with soy protein can significantly decrease the risk associated with elevated glucose release in the blood and a high glycemic index. Further, a study showed that high protein and low carbohydrate snacks before bedtime can be suggested for people with type 2 diabetes to preserve the insulin response [93]. It should be noted that soy protein-based snacks may present a beany flavor, which can affect the sensory properties. Also, the structure and composition of soy protein can change the texture, density, and porosity of snacks.

### 6.3. Application in Pasta Products Formulation

Pasta products are generally processed from wheat using a cold extrusion process temperature (40–50 °C). Single-screw extruders are widely used to manufacture pasta products. Durum wheat semolina is the major ingredient utilized in pasta processing, and it enhances the interaction between the ingredients and improves the physicochemical properties of the dough during processing [94]. The composition of durum wheat semolina rises to a 74–76% dry starch basis and 12–15% protein [95]. The balance between glutenins and gliadins may significantly influence the elasticity and extensibility during dough preparation [96]. Further, raw material formulation, mixing, extrusion and drying conditions, cooling, and stabilization affect the quality of the pasta. The lack of essential amino acids is the drawback associated with the use of durum wheat semolina during pasta processing [97]. To improve the nutritional value of pasta, soy protein can be added during processing. Soy protein-based extrudates can ameliorate the biological functions of pasta and may be recommended to people suffering from gluten intolerance or celiac disease. 

Soy protein isolates and rice flour were mixed and extruded to manufacture gluten-free spaghetti [98]. It was pointed out that a high concentration of soy protein significantly decreased starch retrogradation and increased the eating quality, firmness, and tensile strength. It was also demonstrated that soy protein isolates reduced the cooking time and cooking loss and increased the overall acceptability of pasta. Khatkar et al. [99] used a blend of soy protein concentrate/wheat flour to manufacture instant noodles, and the results showed that the addition of soy protein enhanced the elastic modulus, dough handling, and smoothness. The addition of soy protein improved the springiness, chewiness, gumminess, and resilience and decreased the cooking loss of instant noodles. Further, Bolarinwa and Oyesiji [77] developed a free gluten pasta using soybean flour, rice flour, and cassava starch. It was pointed out that the addition of soy flour enhanced the color and other sensory attributes of the pasta and decreased the cooking time and loss. Soybean flour, sweet potato, and rice flour were used to prepare pasta with improved firmness, water absorption, and low cooking time [100].

The particle size of ingredients, the temperature of water and rehydration method, the distribution of ingredients during mixing, the temperature and pressure during processing, the integrity of the matrix network, and the drying temperature significantly influence a product’s overall acceptability and chemical stability [101]. It was previously reported during pasta manufacturing that soy proteins could interact with wheat proteins, yielding a larger polymer, and denatured soy proteins during heat treatments may crosslink wheat proteins by disulfide linking and then increase the size and significantly decrease the solubility, which in turn enhances the quality of the spaghetti [102]. The concentration of starch in wheat flour ranges from 74 to 76%. The interaction between soy proteins and starches during processing can influence the texture of the products, change the gelatinization characteristics, and significantly decrease the digestibility of starch [103]. The analysis of the influence of soy proteins in pasta formulation and its effect on the spatial arrangements of macromolecules during processing is important to achieve high product quality.

Comparing durum wheat semolina pasta and soy protein-enriched pasta, it was demonstrated that wheat semolina pasta is a rich source of carbohydrates that can enhance glucose release in the blood [104]. Durum wheat semolina pasta is rich in gluten, and De Arcangelis et al. [105] reported that celiac disease is a persistent intolerance resulting from gluten ingestion and can lead to immunologically mediated inflammatory impairment of the small intestinal mucosa. Further, inflammation of the small intestine can cause poor absorption of nutrients such as vitamins and minerals [106]. Apart from its health benefits, soy protein can provide the necessary protein needs when consumed as the only protein source at a level of 0.6 g protein/kg body weight [11].

### 6.4. Application Cereal Breakfast Processing

Breakfast cereals are manufactured from extruded corn, oats, and whole grains. Cereals are a rich source of starch; thus, subjecting starch to high temperatures and pressures generates small structures that are easily digested and absorbed in the small intestine, which can lead to a high glycemic index [92]. Considering the health benefits of soy protein, the incorporation of soy proteins in breakfast cereals is advantageous because it can increase the nutritional value, lower the glycemic index, and generate products with different physicochemical properties and sensory properties. However, a high concentration of soy proteins (nearly 60% *w*/*w*) can significantly decrease the overall acceptability, while 41–47% *w*/*w* showed a good aroma, texture, and overall acceptability [102]

Na-Nakorn et al. [76] developed extruded products using soy protein isolates, dietary fibers, rice, and resistant maltodextrin; they found that 20% soy protein can prevent the swelling of starch granules. Further, findings showed that a concentration of ≥30% soy protein may increase the hardness and stickiness of extruded cereal breakfasts [76].

Substitution of cereals with soy protein during breakfast cereal extrusion is conducted to achieve high protein content and low-calorie products. Soy flour at 10–30% can increase the protein concentration of breakfast cereal to 15–18% [76]. It is important to understand the relationship between product attributes and physicochemical traits in the extrusion system of breakfast cereals. For example, soy flour with a particle size range of 500–700 µm does not significantly affect the water-holding capacity, while soy flour with a particle size of ≤400 µm significantly affects the water-holding capacity and a high soy flour percentage is required in the formulation [34]. The use of a high soy protein concentration in the formulation decreases the porosity and affects the microstructure, texture, and cell size of breakfast cereals, which can change the crispness [107]. It was revealed that high temperature and high pressure at a low feed moisture could enhance the redness and the strong intensity of a soy product’s beany and off-flavor [107].

Extrusion can enhance starch digestibility, and consuming breakfast cereals containing highly digestible starch can cause serious health problems such as diabetes mellitus and increase the risk of heart disease and hypertension [108]. In another study, it was demonstrated that the consumption of high-carbohydrate breakfast cereals promotes a high energy intake and can increase the risk of obesity in children and adults [37]. On the other hand, the consumption of soy protein breakfast cereals can promote energy intake control and prevent the risk of diabetes and other related diseases.

## 7. Conclusions and Future Directions

This study demonstrated that extrusion technology, regardless of the moisture content, influences the conformational characteristics of soy proteins, which may affect the digestibility and availability of specific peptides and amino acids. Extrusion at 25% moisture resulted in the formation of large aggregates via hydrogen bonds; however, extrusion at a moisture of 18% favored the dissociation of soy protein aggregates. Additionally, the dissociation of large aggregates at 18% improved the solubility. Larger aggregates produced at 25% have demonstrated a high hydrophobicity attributed to the aggregation that buries hydrophilic amino acids. The effect of a low moisture extrusion of 8–12% on the quality of the extrudates was investigated. It was demonstrated that products extruded at a low moisture level of 8% were less resilient, had increased air cell size, and were less uniform, which may lead to fragile extrudates during handling. On the other hand, during high moisture extrusion, the amount of water in the matrix positively correlates with the reaction rate. It was pointed out that increasing the moisture content can significantly decrease the viscosity and increase soy protein mobility, resulting in a high reaction rate during extrusion. Decreasing moisture content during extrusion may prevent the ongoing interaction between soy proteins. Temperature is another important variable during high moisture extrusion as it monitors from the melting’s beginning to the endpoint to guarantee melting, conformational change, and high protein texturization. At elevated temperatures, denaturation, polymerization, and aggregation reactions lead to the crosslinking and texturization of soy proteins. The blends of soy proteins and wheat proteins are widely utilized in textured protein and meat analog production. Wheat proteins have demonstrated a high binding capacity, dough-forming capacity, leavening capacity, and viscoelastic behavior. However, the use of wheat proteins in meat analogs is restricted by people with a high sensitivity to gluten. The blends of soy proteins and plant proteins such as pea protein, corn protein, mung bean protein, and peanut protein are used in the formulation of meat analogs to tackle the drawbacks linked with wheat proteins.

With the incorporation of soy proteins or other legume proteins in food formulation, the food industry can address the problems associated with diabetes, obesity, and other cardiovascular diseases. Soy proteins affect molecular biomarkers and enzymes involved in metabolic pathways. The progress of extruded snack processing has been affected by the design and development of low-calorie products with ideal sensory properties. However, it was confirmed that the incorporation of an ideal amount of soy protein in extruded snacks may be one of the effective ways to mitigate problems linked with hypercaloric snacks. Soy proteins may slow the progression of diabetic nephropathy and improve renal function markers. Soy proteins may play a crucial role in cholesterol-lowering, can decrease the level of luminal secretory immunoglobulin A, and can also attenuate the production of intestinal mucin. While the incorporation of legume proteins in food formulation holds great promise, clinical trials are required to comprehensively investigate the health benefits of extruded products containing soy proteins. Also, it is paramount to investigate the minimum concentration of soy proteins required in extruded product formulation to induce biological functions, significantly decrease the amount of calorie intake, and maintain sensory properties. It is also necessary to explore the effect of different snack formulations on starch degradation, starch metabolism, and glucose absorption.

Although soy proteins are used in the reformulation of low-caloric foods, there are still many challenges associated with their chemical composition and functional properties that may influence the quality of extruded products. For example, soy proteins are extracted using different techniques that affect the ratio between 11S and 7S, molecular weight, and solubility, which affect the hydration, unfolding and aggregation rate, and texturization during extrusion. Understanding the correlation between extraction conditions, chemical compositions, functional properties, and extrusion conditions may be beneficial to the development of high-quality soy protein-based extrudates. Therefore, future research should focus on producing soy proteins with the desired 11S and 7S ratio, molecular weight, and solubility depending on the type of extrusion technique and requirements of final product attributes. Additionally, studies on improving texture, chewiness, juiciness, and sensory properties should be further carried out on the resultant products.

## Figures and Tables

**Table 1:** Extrusion of soy protein with or without other ingredients.

Extrusion Conditions						Major Findings	Reference
Composition		Screw Speed	Feed Rate	Temperature	Moisture Content		
Soy Protein Purity	Others						
%	%	RPM	Kg/h	°C	%		
80	0	180	40	110, 130 and 160	18–25	7S and 11S disappeared at 18% Moisture content. 7S and 11S decreased at 130–160 °C. Solubility at 25% moisture extrusion and hydrophobicity at 18% were lowered. At 130–160 °C, the alpha helix decreased.	[34]
90	Volatile compounds 4.14, 3.75 and 3.46 mL	170	2.7	50, 90, 120, 130, 150 and 170	30–40	The addition of volatile compounds decreased the expansion rate at 35% moisture/150 °C and 40% moisture/130 °C. 30% moisture/170 °C favored the reduction of the dough viscosity, allowing greater interactions between the volatiles and the protein matrix.	[27]
40.8–41.4	-	320	2.5	55–95, 70–110, 85–125, 100–140, 110–150	15–27	The nitrogen solubility index increased when extrusion temperature and moisture changed from 150 to 110 °C and 27 to 15 °C, respectively. Gel-forming ability increased when the extrusion temperature changed from 150 to 110 °C. Oil holding capacity improved in samples extruded at 110 °C.	[31]
94.3	Soy flour, Corn flour 7, soy fibers 2, 18	200 and 400	20	30, 75, 120, 130 and 140	20 and 40	Changing the moisture from 20 to 40% decreased the water-holding capacity and the specific volume of the extrudates. Increasing the moisture content decreased the expansion ratio. High screw speed and temperature significantly decreased the whiteness.	[35]
90	-	200	0.3	40, 60, 90, 105, 120, 120 and 90	60	Lightness decreased, and redness increased when the temperature changed from 120–160 °C. Bands near 100 kDa in SPI disappeared after extrusion. 7S disappeared, and 11S was more intense following extrusion.	[36]
90.3–92.8	-	180	0.3	25, 40, 60, 90, 130, 140, and 110 °C	50	The soluble Soy protein isolate portion showed a compact and smooth gel structure, indicating that SSPI alone was unable to create a distinct fibrous structure. High content of insoluble soy protein, the fibrous structures in the extruded SPI became more pronounced.	[37]
67	-	150, 500 and 800	15	40, 60, 100, 100, 100 and 100–160	60	At ≥180 rpm and ≥100 °C high water evaporation and no formation of anisotropic texture. At =180 rpm and 100–132 °C there was a formation of anisotropic texture.	[38]
92	-	175		35, 60, 90, 120, 145, 145, 145, and 120 Cooling at 60 to 20 °C	65	Lowering a cooling die temperature from 60 °C to 40 °C, the cross-fiber structure of samples became richer with a defined orientation. When the cooling temperature dropped from 60 °C to 20 °C, the expansion ratios of textured vegetable decreased significantly.	[39]
90 and 70	-	30–160	1.4	110, 140, 150, 160, 170, 180, and 190	60	Better textural properties and the highest fibrous degree at 160 °C and 30 rpm. Extrusion reduced the ordered secondary structure and increased the disordered structure of soy proteins.	[40]
70	Wheat gluten 0–30, wheat starch 2.7, pumpkin powder 3	400	2.8	20, 50, 80, 110, 150, 180 and 150	66	30% wheat gluten increased the lightness, hardness, chewiness, texturization, and fibrous structure.	[41]
66.5	iota carrageenan (0.75–3)	50	6	60, 135, 125 and 20	60	Iota carrageenan addition did not affect the color. Iota carrageenan improved the hardness and water-holding capacity of the extrudates. 1.5% iota carrageenan has better physical, textural, and sensorial properties.	[42]
90.12	β-glucan 1,2,3,4 and 5	300	-	60, 80,120, 150, 150, and 50	60	The hardness, cohesiveness, and chewiness of the extrudates increased due to β-glucanx, however, at 4% addition, the product attributes decreased. Addition of β-glucan preserved the bands of 7S and 11S due to molecular interaction and crosslinking effect. Large molecular weight bands 50–100 kDa were observed in samples with β-glucan The inclusion of 4% BG changed β-sheet into α-helical structures. This increased α-helical structures and decreased the content of β-turn and β-sheet, promoting the development of a stable and orderly structure of BG and SPI.	[43]
90	Wheat gluten 30% and maize oil 4.6–11%	400	0.3	40, 60, 90, 115, 140, 140, and 140. Cooling at 40 °C	60	oil direct addition approach could not be used to obtain the expected fibrous structure of SPI-WG extrudates O/W emulsion promoted the generation of fibrous structures in SPI-WG extrudates. The fibrous structure could be maintained in SPI-WG extrudates with oil contents up to 8.0%	[44]

**Table 2:** Extruded soy protein in different food systems.

Feed Composition		Extrusion Conditions					Products	Findings	References
Soy Protein	Others	Extruder Type	Feeding	Screw Speed	Temp	Moisture			
%	%		Kg/h	RPM	℃	%			
20	Wheat Gluten 20 and starch 5	Co-rotating twin-screw extruder	6	200	130, 150, and 130	40–50	Meat analogs	Meat analogs at 50% moisture content and 130℃ die Temperature showed texturized properties with the highest water absorption capacity and integrity index.	[70]
80–90	Whole Tomato Powder 5–20%, Tomato Peel Powder 10%	Co-rotating twin screw extruder	3	300	30, 60, 100, and 90	60	Meat analogs	Tomato peel powder led to a soft texture and color improvement. 20% whole tomato powder led to poor textural properties.	[71]
15–60	Mushroom Protein 15–45	Co-rotating twin-screw extruder	2.28	500	50, 80, 120, 140, 140, 120, and 50	65	Meat analogs	Mushroom Protein led to a significant change in darker and brown. The addition of mushroom protein up to 25% showed excellent sensory properties. Mushroom protein decreased the oil and water holding capacity.	[72]
33	Soybean meal 43%, Gluten 24%, Wheat starch 8%, and *Haematococcus pluvialis* (*HP*) (0.25~1.25%) as a colorant	Co-rotating twin-screw extruder	3.2	280	30, 90, 120, 140, 150, 160, and cooling at 60 °C	50	Meat analogs	Low levels of HP promoted the flexible and disordered regions within the protein secondary structure, while excessive HP addition was unfavorable for protein cross-linking. The maximal texturization degree was obtained at 0.75% HP 1% HP had a color similar to fresh beef sirloin.	[73]
10–40	Wheat gluten 10–40, Natural flavor powder 1%	Co-rotating twin-screw extruder	6		20, 50, 80, 150, 140, 100, 80, and 60 °C	50–80	Meat analogs	Soy protein and wheat gluten affected the microstructure of meat analogs. Soy protein isolate and wheat gluten ratio and moisture content affected the flavor retention.	[60]
16–32	Raw Banana	Co-rotating twin-screw extruder	15	200–300	60, 70, and 80 °C	10–20	Snacks	The optimum conditions for the extrudate were found at the screw speed of 200 rpm, barrel temperature of 80 °C, moisture content of 10%, and defatted soy flour content of 32%.	[74]
5–20	Maize grit	Single-screw extruder	100	170	50, 100, and 150	17–24	Snacks	< 20% soybean flour and feed moisture content resulted in snacks with improved nutritional value and physical properties.	[75]
20	Maize Bran 20%, 20% resistant maltodextrin	Twin-screw extruder	0.5	30	70, 90, 90, and 70 °C.	28	Reformulated rice	High concentrations of soy protein increased the hardness. SPI delayed starch gelatinization during cooking.	[76]
10–30	Tapioca Flour 20, rice flour 20–60	Single-screw extruder	-	-	60	10–12	Pasta	(30% soybean protein pasta) had the lowest sensory value. Rice pasta enriched with 15% soybean protein was highly ranked for sensory attributes.	[77]

## Data Availability

No new data were created or analyzed in this study. Data sharing is not applicable to this article.
